# Supplementary material for: Bacillus amyloliquefaciens-9 Reduces Somatic Cell Count and Modifies Fecal Microbiota in Lactating Goats
Source: Mar Drugs. 2021 Jul 21;19(8):404. doi: 10.3390/md19080404 (PMC8400666; doi:10.3390/md19080404)
Supplement: Supplementary file 1 [file marinedrugs-19-00404-s001.zip › Supporting file 1.pdf]

Table S1. TOP 35 Genus in three groups fed with *Bacillus amyloliquefaciens*-9 (BS), *Radix Tetrastigmae* (RS), and control (CS)

Bacterial taxa	Group%			SEM	*P*-value
	CS	BS	RS		
Firmicutes					
Dialister	0.0241	0.0405	0.6381	0.009064237	0.0014*
Blautia	0.1868	0.2608	0.8984	0.009139154	0.8128
unidentified_Ruminococcaceae	2.818	2.8578	2.4198	0.006432995	0.8188
unidentified_Clostridiales	0.558	1.037	0.7313	0.006458744	0.8169
Subdoligranulum	0.0376	0.0799	0.4773	0.005862734	0.8142
unidentified_Erysipelotrichaceae	0.0296	0.0267	0.4163	0.005833614	0.8144
Lachnoclostridium	0.5335	0.6034	0.8126	0.006391016	0.8152
Anaerosporobacter	0.4496	0.464	0.497	0.002814602	0.8163
Butyrivibrio	0.0945	0.6542	0.2021	0.003882475	0.8166
Oscillibacter	0.8704	0.7447	0.6966	0.002477579	0.4675
Megamonas	0.0084	0.0368	0.2121	0.002552855	0.2970
Lactobacillus	0.0004	0.1222	0.0322	0.002415496	0.6077
unidentified_Lachnospiraceae	0.2266	0.3278	0.4364	0.00202981	0.1951
Romboutsia	0.0398	0.0966	0.2102	0.002183952	0.3816
Streptococcus	0.012	0.0668	0.1589	0.002235687	0.5188
Phascolarctobacterium	0.2409	0.5949	0.3159	0.002028095	<0.0001*
Cellulosilyticum	0.0416	0.1887	0.0716	0.001761599	0.2532
Faecalibacterium	0.0208	0.0434	0.1614	0.001734701	0.2936
Anaerostipes	0.027	0.0574	0.1317	0.001250916	0.3154
Butyricicoccus	0.0237	0.0366	0.1201	0.001057539	0.1964
Bacteroidetes					
Bacteroides	3.7902	2.848	3.4889	0.006764765	0.0125*
Alistipes	2.5841	2.82	2.7507	0.007313781	0.8415
unidentified_Prevotellaceae	1.6144	0.3048	0.2599	0.008001492	0.0005*
unidentified_Bacteroidales	0.4722	0.2426	0.2405	0.00279082	0.2442
Alloprevotella	0.3252	0.0114	0.0185	0.003634249	0.2184
Proteobacteria					
Succinivibrio	5.9797	16.4733	13.9385	0.08232045	0.0424*
Ruminobacter	3.1997	0.2962	1.477	0.035418085	0.3128
unidentified_Enterobacteriaceae	0.0551	0.3438	0.3106	0.00478461	0.5131
Mailhella	0.4051	0.4686	0.4041	0.001688546	0.6980
Fibrobacteres					
Fibrobacter	2.2797	0.1574	0.0885	0.017645006	0.0341*
Verrucomicrobia					
Akkermansia	0.5883	1.1	1.9093	0.015213114	0.3113
Actinobacteria					
Bifidobacterium	0.0277	0.0589	0.487	0.004582803	0.1132
Spirochaetes					
unidentified_Spirochaetaceae	0.0336	0.0293	0.0791	0.000910524	0.5403
Euryarchaeota					
Methanobrevibacter	0.3872	0.3514	0.2512	0.002574253	0.6348
unidentified_Bacteria					
Campylobacter	0.2014	0.0725	0.0763	0.001454062	0.2013

*represents significant differences among the three groups.

Table S2. All the OTUs in MEpink generated using a weighted gene co-expression network analysis (WGCNA).

OTU	Taxonomy
OTU_17	*k__Bacteria;p__Firmicutes;c__Clostridia;o__Clostridiales;f__Ruminococcaceae*
OTU_19	*k__Bacteria;p__Bacteroidetes;c__Bacteroidia;o__Bacteroidales;f__Prevotellaceae*
OTU_26	*k__Bacteria;p__Firmicutes;c__Clostridia;o__Clostridiales;f__Ruminococcaceae;g__;s__*
OTU_38	*k__Bacteria;p__Bacteroidetes;c__Bacteroidia;o__Bacteroidales;f__Prevotellaceae;g__;s__*
OTU_48	*k__Bacteria;p__Firmicutes;c__Clostridia;o__Clostridiales;f__unidentified_Clostridiales;g__unidentified_Clostridiales*
OTU_72	*k__Bacteria;p__Bacteroidetes;c__Bacteroidia;o__Bacteroidales;f__Bacteroidaceae;g__Bacteroides*
OTU_78	*k__Bacteria;p__Firmicutes;c__Clostridia;o__Clostridiales;f__Ruminococcaceae*
OTU_99	*k__Bacteria;p__Bacteroidetes;c__Bacteroidia;o__Bacteroidales;f__;g__;s__*
OTU_101	*k__Bacteria;p__Bacteroidetes;c__Bacteroidia;o__Bacteroidales;f__;g__;s__*
OTU_133	*k__Bacteria;p__Bacteroidetes;c__Bacteroidia;o__Bacteroidales*
OTU_136	*k__Bacteria;p__Firmicutes;c__Clostridia;o__Clostridiales;f__Lachnospiraceae;g__Blautia;s__*
OTU_140	*k__Bacteria;p__Bacteroidetes;c__Bacteroidia;o__Bacteroidales;f__Prevotellaceae;g__Alloprevotella;s__*
OTU_163	*k__Bacteria;p__Bacteroidetes;c__Bacteroidia;o__Bacteroidales*
OTU_183	*k__Bacteria;p__Bacteroidetes;c__Bacteroidia;o__Bacteroidales;f__Prevotellaceae;g__unidentified_Prevotellaceae;s__bacterium_P3*
OTU_192	*k__Bacteria;p__Firmicutes*
OTU_196	*k__Bacteria;p__Bacteroidetes;c__Bacteroidia;o__Bacteroidales;f__;g__;s__*
OTU_235	*k__Bacteria;p__Firmicutes;c__Clostridia;o__Clostridiales;f__Ruminococcaceae;g__;s__*
OTU_242	*k__Bacteria;p__Bacteroidetes;c__Bacteroidia;o__Bacteroidales;f__Paludibacteraceae;g__;s__*
OTU_244	*k__Bacteria;p__Tenericutes;c__Mollicutes;o__;f__;g__;s__*
OTU_248	*k__Bacteria;p__Firmicutes;c__Clostridia;o__Clostridiales*
OTU_282	*k__Bacteria;p__Bacteroidetes;c__Bacteroidia;o__Bacteroidales;f__Rikenellaceae;g__;s__*
OTU_285	*k__Bacteria;p__Firmicutes;c__Clostridia;o__Clostridiales;f__Ruminococcaceae;g__unidentified_Ruminococcaceae*
OTU_293	*k__Bacteria;p__Spirochaetes;c__Spirochaetia;o__Spirochaetales;f__Spirochaetaceae;g__;s__*
OTU_308	*k__Bacteria;p__Spirochaetes;c__Spirochaetia;o__Spirochaetales;f__Spirochaetaceae;g__;s__*
OTU_345	*k__Bacteria;p__Bacteroidetes;c__Bacteroidia;o__Bacteroidales;f__Muribaculaceae;g__;s__*
OTU_380	*k__Bacteria;p__Bacteroidetes;c__Bacteroidia;o__Bacteroidales;f__Barnesiellaceae;g__;s__*
OTU_409	*k__Bacteria;p__Firmicutes;c__Clostridia;o__Clostridiales*
OTU_434	*k__Bacteria;p__Bacteroidetes;c__Bacteroidia;o__Bacteroidales;f__Muribaculaceae;g__;s__*
OTU_458	*k__Bacteria;p__Firmicutes;c__Clostridia;o__Clostridiales;f__Ruminococcaceae*
OTU_467	*k__Bacteria;p__Bacteroidetes;c__Bacteroidia;o__Bacteroidales*
OTU_480	*k__Bacteria;p__Firmicutes;c__Clostridia;o__Clostridiales;f__Ruminococcaceae*
OTU_491	*k__Bacteria;p__Firmicutes;c__Clostridia;o__Clostridiales;f__Ruminococcaceae*
OTU_515	*k__Bacteria;p__Bacteroidetes;c__Bacteroidia;o__Bacteroidales;f__Muribaculaceae;g__;s__*
OTU_538	*k__Bacteria;p__Firmicutes;c__Clostridia;o__Clostridiales;f__Ruminococcaceae*
OTU_552	*k__Bacteria;p__Bacteroidetes;c__Bacteroidia*
OTU_555	*k__Bacteria;p__Firmicutes;c__Clostridia;o__Clostridiales;f__Ruminococcaceae;g__;s__*
OTU_587	*k__Bacteria;p__Firmicutes;c__Clostridia;o__Clostridiales;f__Lachnospiraceae;g__unidentified_Lachnospiraceae*
OTU_595	*k__Bacteria;p__Firmicutes;c__Clostridia;o__Clostridiales;f__Lachnospiraceae;g__Roseburia*
OTU_604	*k__Bacteria;p__Firmicutes;c__Clostridia;o__Clostridiales*
OTU_654	*k__Bacteria;p__Firmicutes;c__Erysipelotrichia;o__Erysipelotrichales;f__Erysipelotrichaceae*
OTU_696	*k__Bacteria;p__Firmicutes;c__Clostridia;o__Clostridiales*
OTU_743	*k__Bacteria;p__Firmicutes;c__Erysipelotrichia;o__Erysipelotrichales;f__Erysipelotrichaceae*
OTU_752	*k__Bacteria;p__Firmicutes;c__Clostridia;o__Clostridiales;f__Lachnospiraceae*
OTU_754	*k__Bacteria;p__Firmicutes;c__Clostridia;o__Clostridiales;f__Lachnospiraceae;g__;s__*
OTU_761	*k__Bacteria;p__Proteobacteria;c__Alphaproteobacteria;o__Rhodospirillales;f__;g__;s__*
OTU_782	*k__Bacteria;p__Firmicutes;c__Clostridia;o__Clostridiales;f__Ruminococcaceae*
OTU_835	*k__Bacteria;p__Firmicutes;c__Clostridia;o__Clostridiales;f__Ruminococcaceae*
OTU_838	*k__Bacteria;p__Firmicutes;c__Clostridia;o__Clostridiales;f__unidentified_Clostridiales;g__Anaerovorax*
OTU_898	*k__Bacteria;p__Firmicutes;c__Clostridia;o__Clostridiales*
OTU_900	*k__Bacteria;p__Firmicutes;c__Clostridia;o__Clostridiales;f__Lachnospiraceae;g__Acetitomaculum;s__*
OTU_916	*k__Bacteria;p__Tenericutes;c__Mollicutes;o__Anaeroplasmatales;f__Anaeroplasmataceae;g__Anaeroplasma;s__*
OTU_921	*k__Bacteria;p__Bacteroidetes;c__Bacteroidia;o__Bacteroidales;f__Rikenellaceae;g__Alistipes*
OTU_923	*k__Bacteria;p__Proteobacteria;c__Gammaproteobacteria;o__unidentified_Gammaproteobacteria;f__Burkholderiaceae;g__Sutterella*
OTU_939	*k__Bacteria;p__Firmicutes;c__Clostridia;o__Clostridiales;f__Lachnospiraceae;g__;s__*
OTU_949	*k__Bacteria;p__Firmicutes;c__Clostridia;o__Clostridiales;f__Ruminococcaceae;g__Caproiciproducens;s__*
OTU_993	*k__Bacteria;p__Firmicutes;c__Clostridia;o__Clostridiales;f__Ruminococcaceae;g__unidentified_Ruminococcaceae;s__Clostridiales_bacterium_NK3B98*
OTU_1012	*k__Bacteria;p__Firmicutes;c__Erysipelotrichia;o__Erysipelotrichales;f__Erysipelotrichaceae*
OTU_1032	*k__Bacteria;p__Firmicutes;c__Erysipelotrichia;o__Erysipelotrichales;f__Erysipelotrichaceae;g__Breznakia;s__*
OTU_1047	*k__Bacteria;p__Firmicutes;c__Clostridia;o__Clostridiales;f__Lachnospiraceae;g__Lachnoclostridium;s__[Clostridium]_scindens*
OTU_1091	*k__Bacteria;p__Firmicutes;c__Clostridia;o__Clostridiales;f__Christensenellaceae;g__unidentified_Christensenellaceae*

OTU	Taxonomy
OTU_1116	*k__Bacteria;p__Firmicutes;c__Clostridia;o__Clostridiales*
OTU_1148	*k__Bacteria;p__Firmicutes;c__Clostridia;o__Clostridiales;f__unidentified_Clostridiales;g__unidentified_Clostridiales;s__bacterium_LD2013*
OTU_1368	*k__Bacteria;p__Bacteroidetes;c__Bacteroidia;o__Bacteroidales;f__Muribaculaceae;g__;s__*
OTU_1373	*k__Bacteria;p__Bacteroidetes;c__Bacteroidia;o__Bacteroidales*
OTU_1374	*k__Bacteria;p__Tenericutes;c__Mollicutes*
OTU_1408	*k__Bacteria;p__Firmicutes;c__Clostridia;o__Clostridiales;f__Eubacteriaceae;g__Pseudoramibacter;s__*
OTU_1410	*k__Bacteria;p__Proteobacteria;c__Gammaproteobacteria;o__Enterobacteriales;f__Enterobacteriaceae*
OTU_1427	*k__Bacteria;p__Firmicutes;c__Erysipelotrichia;o__Erysipelotrichales;f__Erysipelotrichaceae;g__Candidatus_Stoquefichus*
OTU_1431	*k__Bacteria;p__Firmicutes;c__Clostridia;o__Clostridiales;f__Lachnospiraceae*
OTU_1449	*k__Bacteria;p__Actinobacteria;c__Coriobacteriia;o__Coriobacteriales;f__Eggerthellaceae;g__Senegalimassilia;s__*
OTU_1472	*k__Bacteria;p__Proteobacteria;c__Deltaproteobacteria;o__Desulfovibrionales;f__Desulfovibrionaceae;g__Bilophila*
OTU_1486	*k__Bacteria;p__Firmicutes;c__Clostridia;o__Clostridiales;f__Peptococcaceae;g__;s__*
OTU_1487	*k__Bacteria;p__Firmicutes;c__Clostridia;o__Clostridiales;f__Ruminococcaceae*
OTU_1489	*k__Bacteria;p__Firmicutes;c__Bacilli;o__Lactobacillales;f__Leuconostocaceae;g__Weissella;s__Weissella_cibaria*
OTU_1503	*k__Bacteria;p__Bacteroidetes;c__Bacteroidia;o__Bacteroidales;f__Rikenellaceae;g__;s__*
OTU_1532	*k__Bacteria;p__Verrucomicrobia;c__Verrucomicrobiae;o__Opitutales;f__Puniceicoccaceae*
OTU_1545	*k__Bacteria;p__Firmicutes;c__Clostridia;o__Clostridiales;f__Ruminococcaceae;g__unidentified_Ruminococcaceae;s__Clostridiales_bacterium_canine_oral_taxon_123*
OTU_1559	*k__Bacteria;p__Lentisphaerae;c__Lentisphaeria;o__Victivallales;f__Victivallaceae*
OTU_1563	*k__Bacteria;p__Firmicutes;c__Clostridia;o__Clostridiales;f__Christensenellaceae;g__;s__*
OTU_1632	*k__Bacteria;p__Proteobacteria;c__Gammaproteobacteria;o__Pasteurellales;f__Pasteurellaceae;g__Actinobacillus;s__Bisgaard_Taxon_10*
OTU_1649	*k__Bacteria;p__unidentified_Bacteria;c__unidentified_Bacteria;o__unidentified_Bacteria;f__unidentified_Bacteria;g__Candidatus_Saccharimonas;s__*
OTU_1675	*k__Bacteria;p__Firmicutes;c__Clostridia;o__Clostridiales;f__Lachnospiraceae;g__;s__*
OTU_1687	*k__Bacteria*
OTU_1692	*k__Bacteria;p__Bacteroidetes;c__Bacteroidia;o__Bacteroidales;f__Prevotellaceae*
OTU_1718	*k__Bacteria;p__Firmicutes;c__Clostridia;o__Clostridiales;f__Ruminococcaceae*
OTU_1775	*k__Bacteria;p__Tenericutes;c__Mollicutes;o__;f__;g__;s__*
OTU_1900	*k__Bacteria;p__Firmicutes;c__Clostridia;o__Clostridiales;f__Lachnospiraceae*
OTU_1988	*k__Bacteria;p__Actinobacteria;c__Coriobacteriia;o__Coriobacteriales*
OTU_2026	*k__Bacteria;p__Firmicutes;c__Clostridia;o__Clostridiales;f__Ruminococcaceae;g__Oscillibacter;s__*
OTU_2054	*k__Bacteria;p__Firmicutes;c__Clostridia;o__Clostridiales;f__Ruminococcaceae;g__;s__*
OTU_2088	*k__Bacteria;p__Proteobacteria;c__Alphaproteobacteria;o__Rhodospirillales;f__;g__;s__*

Table S3. Nutrient compositions of the basal diet

Items	Experimental diets
Ingredient (of DM basis %)	
Whole corn silage	34.3
Alfalfa hay	20.1
Corn	27.8
Wheat bran	12.6
Soya bean meal	3.2
Premix	1.2
NaCl	0.3
$NaHCO_3$	0.5
Total	100
Nutrient composition	
Dry matter (%)	47.9
Crude protein (%)	10.4
Ether extract (%)	3.4
Crude fibre (%)	18.5
Neutral detergent fibre (%)	44.6
Acid detergent fibre (%)	23.7
Ash (%)	4.1
