# Supplementary material for: Bacillus amyloliquefaciens-9 Reduces Somatic Cell Count and Modifies Fecal Microbiota in Lactating Goats
Source: Mar Drugs. 2021 Jul 21;19(8):404. doi: 10.3390/md19080404 (PMC8400666; doi:10.3390/md19080404)
Supplement: Supplementary file 1 [file marinedrugs-19-00404-s001.zip › supporting file 2.pdf]

Figure S1. CCA and RDA analyses of milk yield and milk compositions in dairy goats after feeding with *Bacillus amyloliquefaciens*-9 (BS), *Radix Tetrastigmae* (RS) and control (CS).

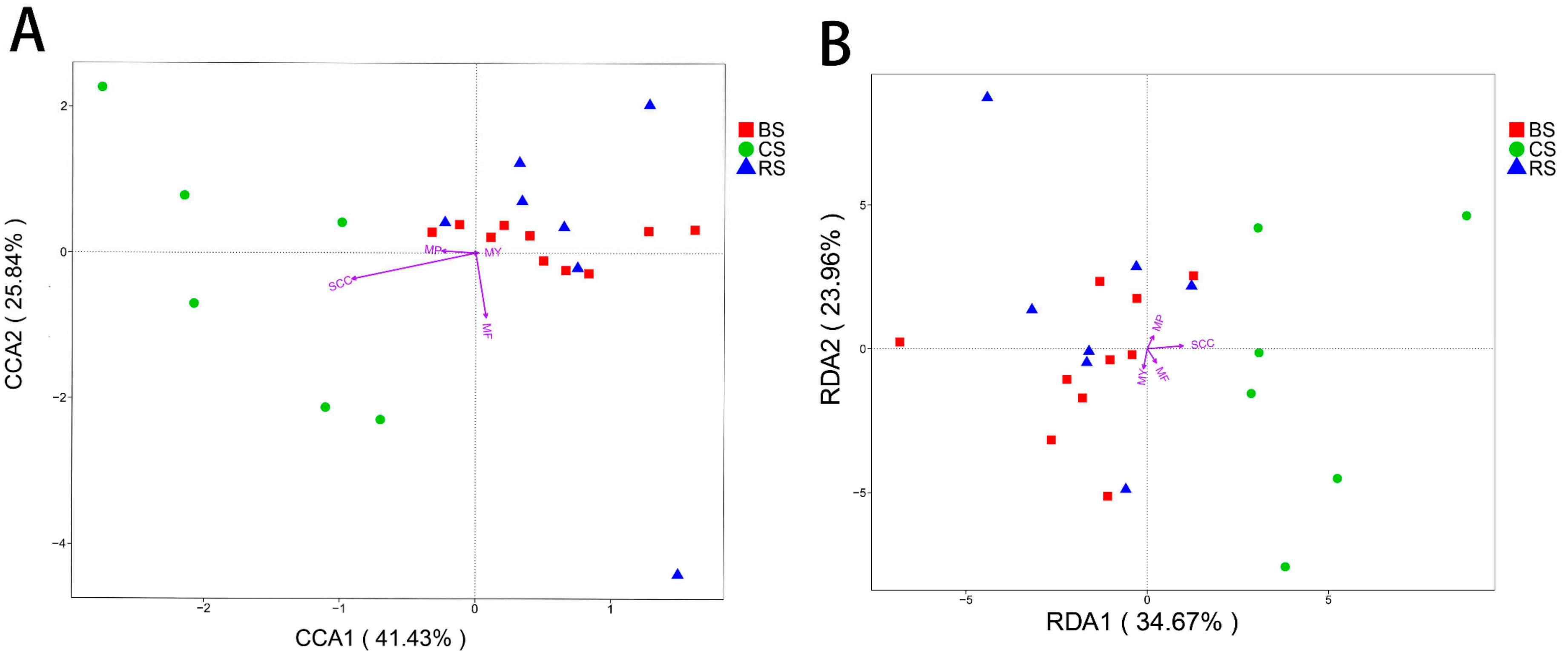

Canonical correlation analysis (CCA) and Redundancy analysis (RDA) were performed at the genus level by using the vegan package in R. CCA and RDA analyses proved the interaction among milk yield, milk protein, milk fat, SCC and microbiota. (A) CCA anlysis. (B) RDA analysis. MY: milk yield; MP: milk protein content; MF: milk fat content; SCC: somatic cell counts.
